# Herpes Zoster Infection as a Presentation for Hidden Diabetes Mellitus

**DOI:** 10.7759/cureus.7363

**Published:** 2020-03-22

**Authors:** Samih A Odhaib, Abbas Mansour

**Affiliations:** 1 Adult Endocrinology, Faiha Specialized Diabetes, Endocrine and Metabolism Center, College of Medicine, University of Basrah, Basrah, IRQ

**Keywords:** herpes zoster, diabetes mellitus, cell-mediated immunity, antiviral

## Abstract

The incidence of undiagnosed diabetes mellitus (DM) is high among patients with herpes zoster (HZ) due to complex immune defects. The DM and HZ affect each other's course aggressively.

We introduced three cases of HZ for two men and one woman who were nondiabetic at presentation. Later on, their treating physicians diagnosed them with DM with different degrees of severity of hyperglycemia. They referred the three patients to us for a second opinion and for managing their DM in the presence of HZ. We managed them according to their glycemic figures. The patients were asymptomatic at different follow-up visits.

The innate immune responses are lower in patients with DM, which is not enough for cutaneous protection during the reactivation of the dormant varicella-zoster virus (VZV). HZ and post-herpetic neuralgia (PHN) show an aggressive course in patients with DM and reduce the patient's quality of life. We illustrated three quiescent risk factors for our patients, in addition to undiagnosed DM, prior statin use, and comorbidity with coronary artery disease (CAD) and thyroid disease. These risk factors might affect the immunomodulatory responses and proinflammatory cytokines in various degrees. The management of patients with HZ and underlying DM is challenging. The therapy relies on antiviral medications for infection control, pain control, and a specific management plan for DM in which premixed insulin and metformin are the main components.

Nondiabetic patients with acute HZ infection, whatever the severity, need to be screened for diabetes and/or hyperglycemia at the baseline interview and on frequent intervals thereafter to diagnose possible underlying DM.

## Introduction

Herpes zoster (HZ) infection (shingles) occurs due to reactivation of a previous infection with varicella-zoster virus (VZV), due to decreased VZV-specific cell-mediated immunity (CMI), with aging or in individuals with immunosuppressive disorders [1]. Usually, a latency period (which reflects the host-virus interaction) of several years follows and then replication of the virus occurs [2-3].

Subsequently, VZV trek along the affected sensory nerves to the skin and induces the distinctive painful vesicular rash, following a dermatomal pattern, which does not cross the midline. The typical presentation of HZ is a painful unilateral vesicular dermatomal rash lasting two to four weeks. Constant or episodic tingling, itching, and/or pain precede the outbreak by two to three days [2].

The diagnosis of HZ is mostly clinical, with occasional direct antigen and/or antibody detection for cases with atypical rashes [2, 4].

The incidence of undiagnosed diabetes mellitus (DM) is high among HZ patients, which may be due to impairment of CMI, phagocytosis, and opsonization, with intact humoral immunity [5-6]. Although HZ deteriorates glycemic control, the latter does not correlate with the severity of impaired CMI [6-7].

The highly prevalent DM increased the risk of HZ by 20%; yet, the current guidelines do not recommend DM screening in HZ [6, 8]. However, given such a high prevalence and the quiescent DM picture in HZ patients, glucose levels must be screened at the time of diagnosis of HZ and repeated one week later to exclude stress hyperglycemia [5, 9-10].

## Case presentation

Case 1

**Figure 1:**
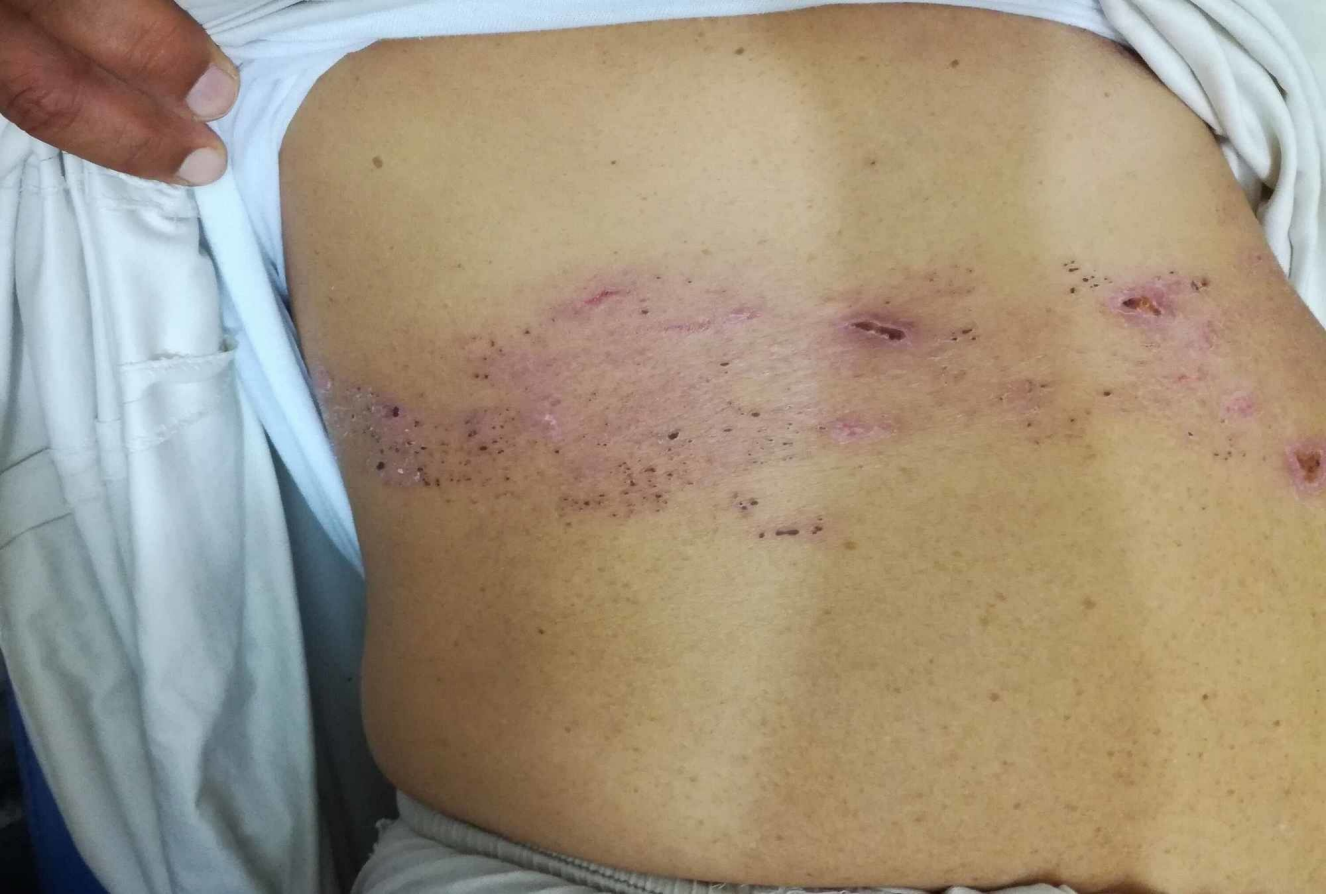
Image of a 56-year-old man with herpes zoster infection on Day 11 after antiviral treatment He was discovered to have undiagnosed diabetes mellitus and was started on insulin therapy.

This 56-year-old nonsmoker, nondiabetic male with hypertension and coronary artery disease (CAD) developed a severe form of an eruptive pruritic rash that involved the right subchondral area that was suggestive of a clinical diagnosis of HZ infection (shingles) (Figure 1).

His baseline investigations were within normal ranges. He had a noncontributory past medical history with no medical condition that might compromise his immune status, and he could not recall a primary chickenpox infection. His medication history included metoprolol, 100 mg, atorvastatin, 20 mg, and acetylsalicylic acid, 100 mg daily. 

Over the following 10 days, he complained of excessive thirst and failure of pain relief on Gabapentin®, 600 mg daily, with oral tramadol, 50 mg daily. He consulted his dermatologist again and was found to have hyperglycemia with a glucose level of 398 mg/dl. The dermatologist referred him for a second opinion. Repeat investigations ensured hyperglycemia and glycated hemoglobin (HbA1c) of 8.9%. We started him on premixed insulin therapy, 30 IU, and metformin, 2,000 mg daily, and kept the same dose of his pain medications. Four months later, his HbA1c was 7.1%. We advised him to rely on the same treatment for six months, after which he presented to the clinic with optimal glycemic control and an HbA1c around 7%.

Case 2

This 47-year-old nondiabetic, nonsmoker female presented with a severe form of HZ infection that involved different dermatomes of the right upper limb from the axilla to the hand, which was itchy, blistering, and eruptive. She denied any history of immune compromising illness or drugs. Her past medical and drug history, as well as the general examination, were noncontributory. Her baseline investigations were in the normal range.

Her treating physician initiated a two-week course of local and systemic antiviral acyclovir with the use of oral paracetamol, 2,000 mg, and Gabapentin, 600 mg, to control the pain. During her second visit to her physician after completion of treatment, she was pain-free with proper healing of the lesions. Unfortunately, one month later, she presented with poorly controlled post-herpetic neuralgia (PHN) and was found to have a fasting hyperglycemia of 198 mg/dl. Her physician referred her for a second opinion.

**Figure 2:**
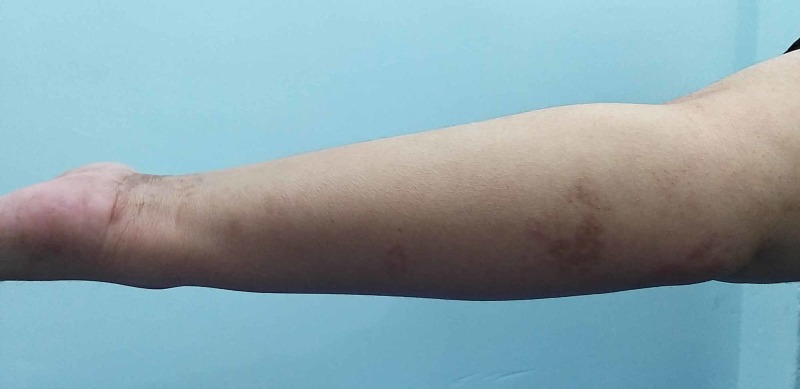
Image of the right forearm of a 47-year-old woman with severe post-herpetic neuralgia that developed six weeks after initial optimal therapy for her shingles The hyperpigmented areas represent the sites of the initial eruptive lesions. She was discovered to have undiagnosed type 2 diabetes mellitus and Hashimoto thyroiditis and was started on levothyroxine and insulin therapy.

**Figure 3:**
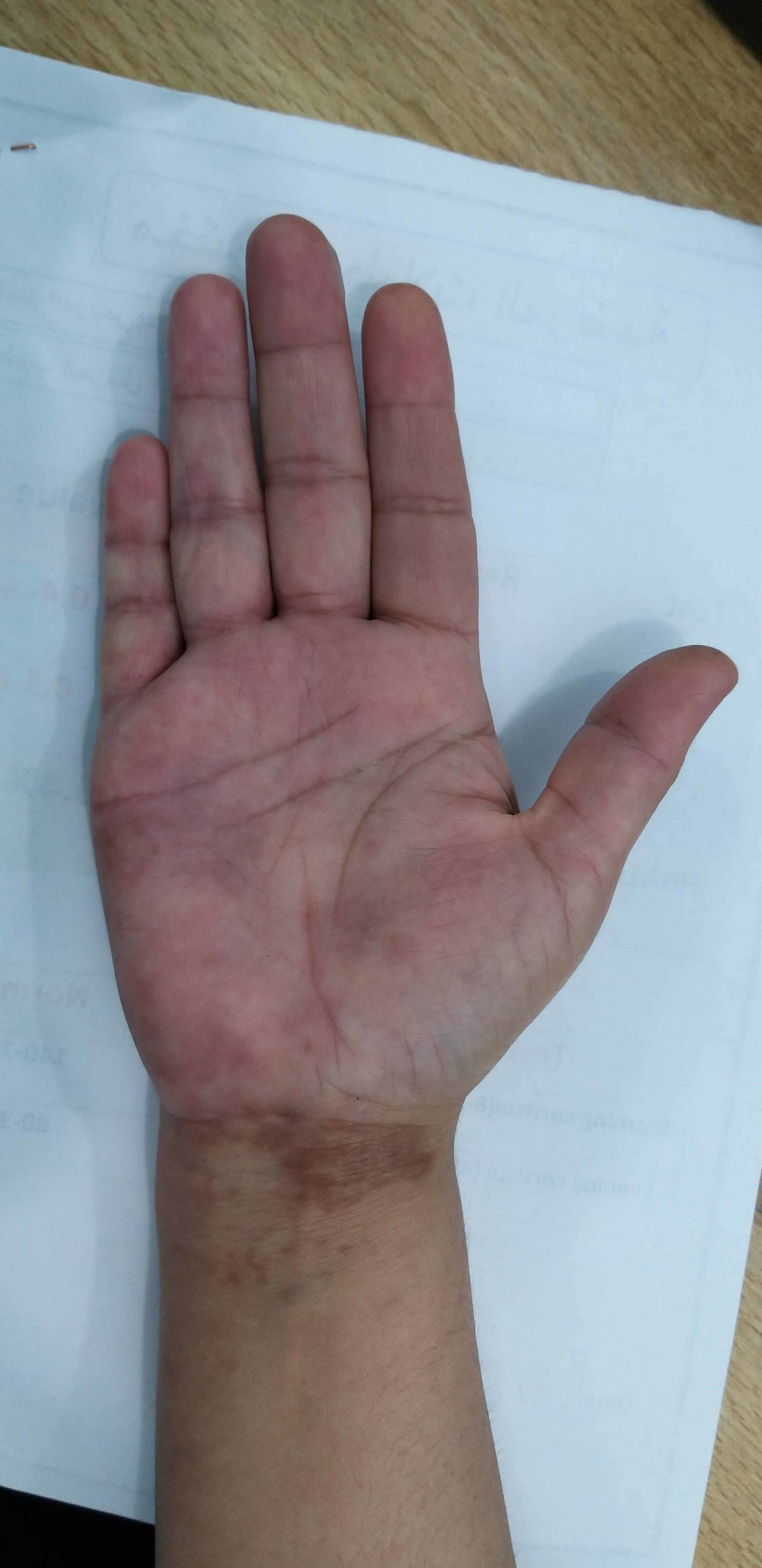
Image of the right hand of the woman (Case 2) demonstrated hyperpigmented areas that involved the palm and represented the sites of previous herpes zoster infection

Figures 2-3 showed many hyperpigmented areas along the whole right upper limb at the site of the recent HZ six weeks earlier. Repeat investigation revealed a fasting plasma glucose (FPG) of 179 mg/dl, HbA1c of 10.6%, negative for antibodies to glutamic acid decarboxylase (anti-GAD), normal C-peptide, thyroid-stimulating hormone (TSH) of 53 mIU/ml, free thyroxine (FT4) of 0.09 pg/ml, and thyroid peroxidase (TPO) antibodies of 387 IU/ml. We diagnosed her as a case of type 2 diabetes mellitus (T2DM) and Hashimoto thyroiditis. We started her on levothyroxine, 100 mcg, in the early morning, insulin Mixtard®, 30 IU, and metformin, 2,000 mg, with an urgent referral to the pain clinic. Six weeks later, she had an HbA1c of 8.6%, with a TSH of 7.9 mIU/ml, and FT4 of 1.09 pg/ml with full resolution of the signs and symptoms of HZ. We advised her to keep the same treatment doses and to gradually quit the Gabapentin, 600 mg, that was prescribed by the pain specialist. She was scheduled for a follow-up visit within four to six months later.

Case 3

A 68-year-old nondiabetic, ex-smoker male presented to the dermatologist with a severe form of shingles involving the left subchondral area of the abdomen that extended to the back on the same side in a scattered, irregular pattern within the same dermatome, with evidence of scratch marks. Initial investigations and past medical history were noncontributory.

**Figure 4:**
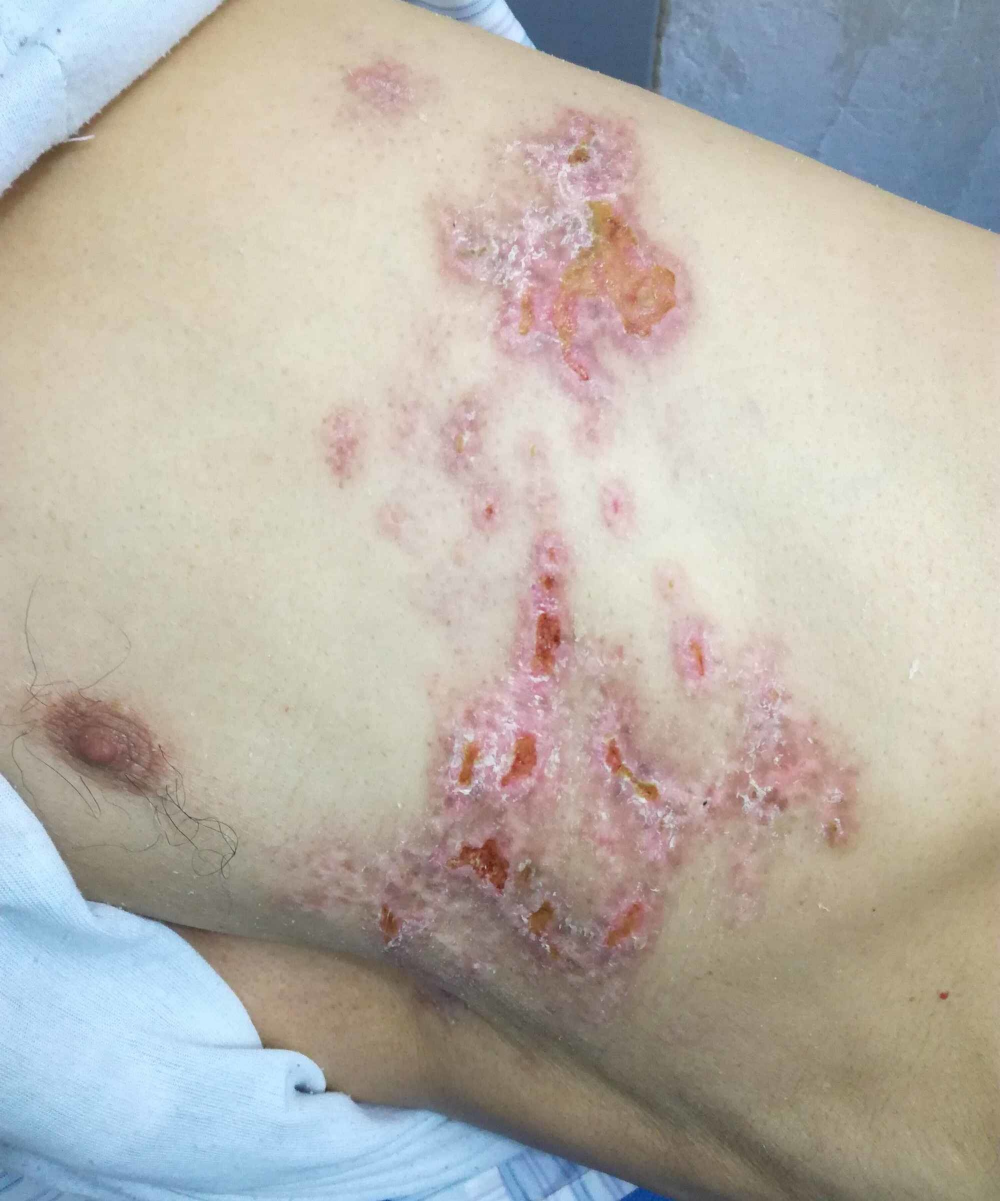
Image of a 68-year-old man with shingles of the left subchondral area on Day 22 of his illness The patient was discovered to be diabetic accidentally, given the resolution of the suboptimal lesions and improper pain control. We started him on insulin therapy with a referral to the pain clinic for escalated pain management.

Three weeks after the initial diagnosis with HZ, his response to the local and systemic antiviral therapy was suboptimal, inadequate pain control, and poor healing. New investigations revealed hyperglycemia (407 mg/dl). His dermatologist referred him for a second opinion. Figure 4 demonstrates an active HZ infection at presentation to our clinic. Repeat investigations revealed an FPG of 198 mg/dl, two-hour postprandial glucose was 317 mg/dL, and HbA1c of 7.9%.

We initiated him on insulin Mixtard, 40 IU, and metformin 2,000 mg daily, with an urgent referral to the pain clinic for escalation of his pain regimen of Gabapentin, 600 mg, and oral tramadol, 50 mg daily. He needed about 11 weeks for full resolution of the signs and symptoms of HZ, after which we kept the same treatment regimen, apart from decreasing the dose of Gabapentin down to 300 mg on an alternate-day basis and to stop the oral tramadol. Six weeks later, we stopped his pain medications and decreased the insulin dose to 15 IU twice daily, given his optimal glycemic control and HbA1c of 6.5%. We scheduled him for a six-month follow-up for diabetes management.

## Discussion

The incidence of undiagnosed DM is high among patients with HZ due to impairment of immunity in different types of DM [5, 10-12]. The innate immune responses by polymorphonuclear cells and monocytes/macrophages are lower in patients with DM, with inadequate cutaneous reaction to the interferon-alpha which is important to cease the VZV spread in the skin [1, 5-6].

Diabetes can affect VZV antigen-antibody response that protects the host by neutralization of infectious VZV at sites of primary infection on reexposure to the virus by contact with individuals who have HZ, which is vital in the HZ recurrence in diabetic patients [1].

The patients with HZ infection may show different levels of severity of the disease and various types of neurologic complications, depending on the site of the nerves involved, whether peripheral or cranial, degree of immunosuppression, and comorbidities [2]. Recent studies demonstrated an effect of racial and gender differences, i.e., Caucasian more than African-American and women more than men, to acquire HZ infection [13-14].

HZ shows an aggressive course in patients with DM, with higher health-related resource consumption, poor glycemic control, and reduced quality of life. This will reflect negatively on the number of outpatient visits, hospitalizations, and medication for diabetes after the HZ episode, as well as an increase in HbA1c levels [3, 6].

There are inconclusive results regarding the role of severe immunosuppression as a risk factor for PHN which can be seen in about 10% - 50% of patients with HZ, especially in diabetic individuals [2, 11-12, 15]. The risk of PHN increases with increasing age (particularly over the age of 50) and is more significant in persons with severe pain at the onset of HZ or a severe rash with a large number of lesions [2-3, 12]. 

Hyperglycemia may predict future PHN [3, 11]; yet, it does not correlate with the severity of CMI impairment in patients with HZ or PHN [7].

In Case 1, there were two risk factors (in addition to undiagnosed diabetes, the prior statin use, and the comorbidity with CAD) that were reported as risk factors in different studies which illustrated an inconclusive association between the prior statin use and the emergence of HZ whatever the dose was [16-17]. The immunomodulatory effect is the basis of the association through diminished proinflammatory cytokines, such as tumor necrosis factor-α, interleukin (IL)-1β, IL-6, and IL-8, that depresses T-cell activation by antigen-presenting cells, increased T-cell apoptosis, and reduced chemotactic effects of neutrophils [16].

On the other hand, there was an escalated risk of HZ occurrence in diabetic patients comorbid with CAD and associated microvascular disorders. Patients with CAD demonstrated a compromised adaptive immune response in CD4+T and CD8+T-cells [14].

For Case 2, an abnormal thyroid hormone level might represent an additional risk factor for HZ reactivation. Ajavon et al. suggested that thyroid hormone levels may play a critical role in the reactivation of the dormant neurotropic VZV, similar to its effect on the other neurotropic virus from the same α herpes virus family, herpes simplex virus-1 (HSV-1), which shares a high degree of genome homology [18]. Ajavon et al. revealed a triple increase in HZ reactivation, not chickenpox (VZV primary infection), occurrence in patients with thyroid hormone disturbance. 

The presence of these risk factors and comorbidities, with an inadequate immune response in patients with DM and HZ, imposes a further challenge on the management of our three patients, although the treatment anchors on infection and pain control with adequate glycemic management.

Early antiviral therapy (within 72 hours after the onset of rash) is the mainstay of treatment in persons with complications of HZ or at increased risk of complications, such as elderly and diabetic persons [2]. Antiviral medications deter viral replication and shedding, mitigate the severity of neurologic signs, and result in better healing of the lesions [9].

Valacyclovir or famciclovir are preferred over acyclovir due to reduced dosing frequency and higher levels of antiviral drug activity [2]. Guidelines suggest a combination of antiviral therapy to reduce pain and other complications of HZ [8].

We referred the three patients to a pain clinic for adequate HZ pain control. Analgesia, membrane stabilizers (pregabalin, gabapentin), and occasional tricyclic antidepressants TCA (amitriptyline) were prescribed by the pain specialist as suggested by guidelines [8-9]. It is controversial to use any corticosteroid and is advisable to avoid its use in persons with diabetes [9].

Ke et al. showed that even oral diabetic medications might increase the risk of HZ emergence and recurrence by unknown mechanisms [14]. This is why we adopted the opinion of Kalra et al., who suggested the use of premixed insulin twice daily, to be the base of our treatment for the DM in our patients in order to have a rapid and sustained glycaemic control that was necessary for optimal management of the viral infection [9].

Insulin has rapid action with minimal drug-drug interactions, an easy dose adjustment, and low risk of hypoglycemia if used judiciously. The data are sparse regarding the preferred glucose-lowering regimen of choice in HZ with DM or stress hyperglycemia [9]. Recent studies have demonstrated an increased risk for acquiring HZ in patients with established DM when they used thiazolidinediones, alpha-glucosidase inhibitors, and dipeptidyl peptidase-4 (DPP-4) inhibitors which appear to increase the risk of HZ, whereas metformin and sulphonylureas did not [3, 14].

Further protection for affected persons may be acquired by vaccination against HZ/PHN to reinforce the CMI against the latent VZV, impeding its reactivation and the associated complications. The vaccine prevents effectively against HZ and PHN and also reduces the severity of the HZ symptoms [2, 6, 19].

## Conclusions

Nondiabetic patients with acute HZ infection, whatever the severity, need to be screened for diabetes and/or hyperglycemia at the baseline interview and at frequent intervals thereafter to diagnose possible underlying diabetes mellitus.
